# Second harmonic generation on crystalline organic nanoclusters under extreme nanoconfinement in functionalized silica–benzil composites

**DOI:** 10.1038/s41598-023-37147-4

**Published:** 2023-06-19

**Authors:** Houda El Karout, Yaroslav Shchur, Anatoliy Andrushchak, Bouchta Sahraoui, Robert Wielgosz, Olha Kityk, Jarosław Jędryka, Yurii Slyvka, Andriy V. Kityk

**Affiliations:** 1 University of Angers, LPhiA, SFR MATRIX, 2 Bd. Lavoisier, 49045 Angers Cedex 01, France; 2 Institute for Condensed Matter Physics, 1 Svientsitskii str., Lviv, 79011 Ukraine; 3 Lviv National Polytechnic University, 12 S. Bandery str., Lviv, 79013 Ukraine; 4 University of Angers, MOLTECH-Anjou-UMR CNRS 6200, SFR MATRIX, 49000 Angers, France; 5 Energia Oze Sp. z o.o., ul. Czȩstochowska 7, 42-274 Konopiska, Poland; 6 Faculty of Electrical Engineering, Częstochowa University of Technology, Al. Armii Krajowej 17, 42-200 Częstochowa, Poland; 7 Department of Inorganic Chemistry, Ivan Franko National University of Lviv, Kyryla i Mefodiya Str. 6, Lviv, 79005 Ukraine

**Keywords:** Materials for optics, Nanoscale materials, Optical materials and structures, Nonlinear optics

## Abstract

We demonstrate a series of organic–inorganic nanocomposite materials combining the mesoporous silica (*PS*) and benzil (*BZL*) nanocrystals embedded into its nanochannels (6.0–13.0 nm in diameter) by capillary crystallization. One aims to design novel, efficient nonlinear optical composite materials in which inactive amorphous host *PS*-matrix provides a tubular scaffold structure, whereas nonlinear optical functionality results from specific properties of the deposited guest *BZL*-nanocrystals. A considerable contraction of the * BZL* melt during its crystallization inside the silica nanochannels results in a formation of the texture consisting of (221)- and (003)-oriented *BZL* nanoclusters (22 nm in length), separated by voids. Specificity of the textural morphology similarly to the spatial confinement significantly influences the nonlinear optical features of composite *PS*:*BZL* materials being explored in the second harmonic generation (SHG) experiment. The light polarization anisotropy of the SHG response appears to be considerably reduced at channel diameters larger than 7 nm apparently due to the multiple scattering and depolarization of the light on randomly distributed and crystallographically oriented *BZL*-nanoclusters. The normalized SHG response decreases nonlinearly by more than one order of magnitude as the channel diameter decreases from 13.0 to 6.0 nm and vanishes when spatial cylindrical confinement approaches the sizes of a few molecular layers suggesting that the embedded *BZL* clusters indeed are not uniformly crystalline but are characterized by more complex morphology consisting of a disordered SHG-inactive amorphous shell, covering the channel wall, and SHG-active crystalline core. Understanding and controlling of the textural morphology in inorganic–organic nanocrystalline composites as well as its relationships with nonlinear optical properties can lead to the development of novel efficient nonlinear optical materials for the light energy conversion with prospective optoelectronic and photonic applications.

## Introduction

Over the last decade, nanocomposite materials have become the subject of great scientific interest and advanced technological applications. A challenge in designing novel functional materials usually lies in the development of nanocomposites with favored and/or controlled mechanical, physical and chemical properties. In relevant technologies, the individual components can be altered and tailored in many ways aiming at utilizing their desired properties to the resultant nanocomposite structure as a whole. As heterogeneous multiphase formations, they can be fabricated combining appropriate organic and inorganic materials in various nanoscale morphologies such as e.g., carbon nanotubes^1–4^ and nanoribbons^5–7^, graphene nanoplatelets^8–10^, carbon, perovskite and semiconductor quantum dots^11–13^, nanoparticles and nanospheres^14–16^, nanowires^17–21^, organic dyes^22^, nanocrystals^23–25^, isotropic liquids^26^ or anisotropic liquid crystal materials^27–30^ being incorporated into organic or inorganic liquids or host solid media such as e.g., polymers or inorganic nanoporous materials. Among the latter ones, mesoporous silica, *p*SiO$_2$ (hereafter *PS*)^31^, likewise the mesoporous silicon Si^32–34^ and alumina *p*Al$_2$O$_3$ (anodic aluminum oxide, AAO)^35^ may be considered as most advanced host matrices for many nanocomposite technologies. Specific morphology of *PS* membranes results from the technology of their fabrication based on unidirectional etching process of *p*-doped silicon with its subsequent oxidation. Thus, relevant mesoporous matrices consist of an array of nanochannels arranged in parallel. Due to this morphological feature, *PS* membranes are characterized by macroscopic dielectric and optical anisotropy which can be easily tuned by filling the nanochannels with both isotropic liquids^26,36^, anisotropic liquid crystals^27,28,37–39^ or by depositing solid state amorphous or crystalline materials in them. In such a functionalized composite material, a scaffold amorphous structure of *PS*-template provides a mechanical robustness whereas its functionalization results from specific properties of a deposited guest material^40^. Importantly, interfacial interaction and spatial confinement in many cases lead to crystalline textures^23,24^ or molecular ordering of confined liquid crystals^27–29^ with a specific phase behavior and physical–chemical properties compared to unconfined bulk systems.

**Figure 1:**
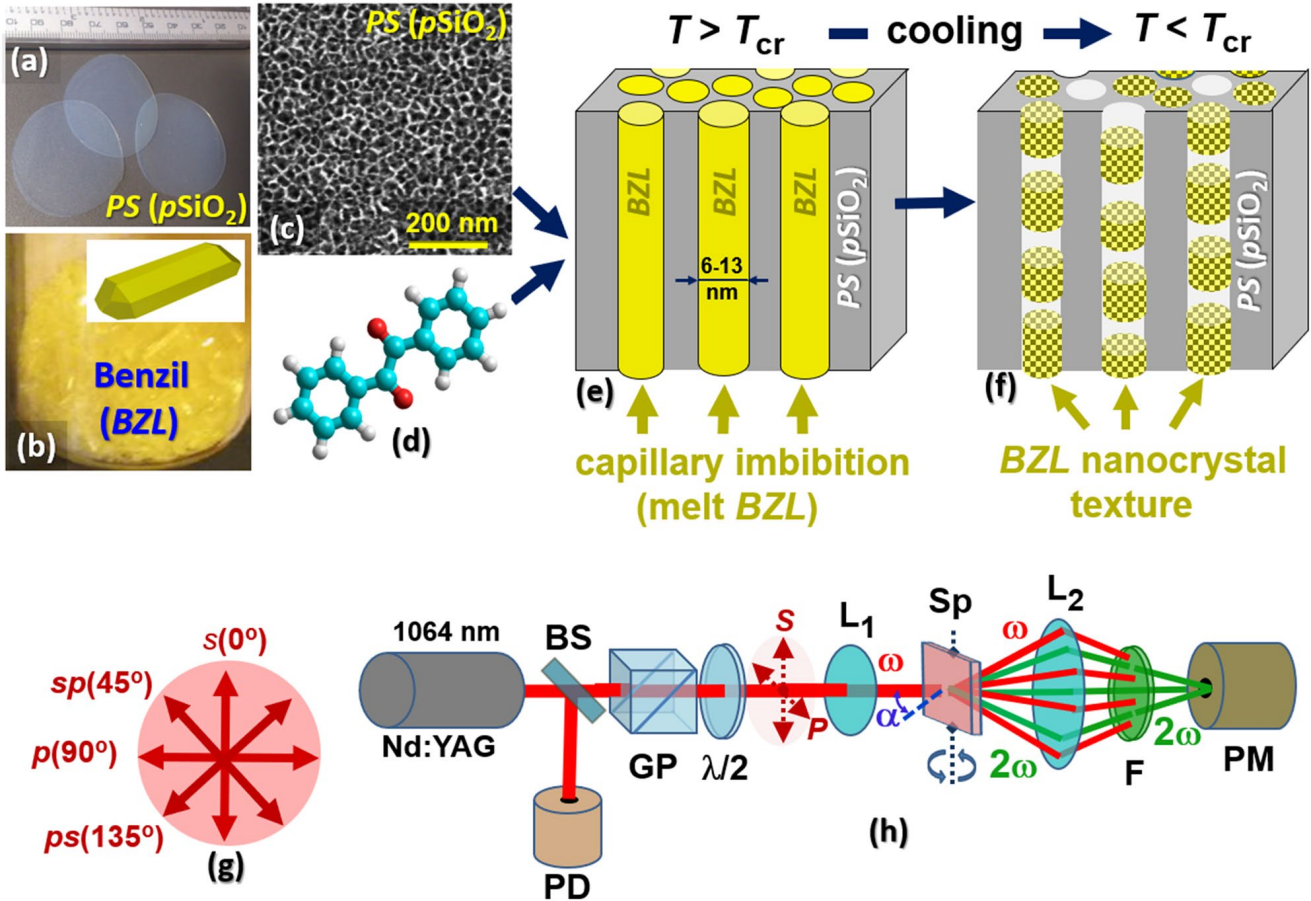
Synthesis and nonlinear optical measurements of silica–benzil (BZL:*p*SiO$_2$) nanocomposites. Mesoporous silica (*p*SiO$_2$) membranes (**a**), obtained by oxidation of electrochemically etched mesoporous silicon, constitute the host scaffold in the composite technology, while crystalline benzil (BZL) (**b**) represents the guest material ensuring non-linear optical functionality of the resulting composite material. (**c**) SEM image of top side of *p*SiO$_2$ membrane. (**d**) BZL molecular structure. *p*SiO$_2$ membranes were completely filled with BZL melt via capillary imbibition at about 105 $^{\circ }$C (**e**). A further slow cooling through the crystallization point ($T_{cr}$ = 96 $^{\circ }$C ) leads to the formation of BZL nanocrystals inside the silica channels (**f**). (**h**) Sketch of the nonlinear optical setup (SHG experiment): Nd:YAG laser ($\lambda =$ 1064 nm, $E_p=$ 100 $\upmu$J, $\tau =$ 30 ps); $\lambda /2$, half-wave plate, *GP* Glan polarizer, $L_1$ *and* L$_2$ lenses, *Sp* measured sample, *F* interference filter (532 nm), *PD* photodiode, *PM* photomultiplier. The rotating half-wave plate serves to set a light polarization direction, (**g**) shows four fixed light polarization directions (*s*, *sp*, *p* or *ps*) used in angular ($\alpha$-scans) SHG measurements.

In this paper, we present a series of organic–inorganic nanocomposites representing mesoporous silica with benzil ((C$_6$H$_5$CO)$_2$, hereafter *BZL*) nanocrystals embedded in nanochannels. Bulk *BZL* represents a yellow-colored molecular crystal (Fig. 1a) whose molecules consist of two phenyl rings and two CO groups, see Fig. 1b. At room temperature, it is characterized by the hexagonal lattice with trigonal space group P3$_{1,2}$21^41,42^. In a symmetry aspect, *BZL* crystal structure is identical with the crystalline quartz ($\alpha$-phase), i.e., both natural and synthetic mineral known for its wide technological applications^43^. Due to its noncentrosymmetric crystalline symmetry (point group 32), *BZL* similarly to the crystalline quartz, is characterized by piezoelectricity^44^ as well as by specific parametrical and nonlinear optical properties including linear electrooptics (Pockels effect) and second order optical nonlinearity. In composite performance, these functionalities may be added to an amorphous *PS*-matrix which is macroscopically centrosymmetric, and the relevant effects are symmetry forbidden. The efficiency of *BZL* crystals as second-order nonlinear optical material has already been particularly demonstrated in earlier study^45^ which is a characteristic feature for many organic materials containing isocyclic and/or heterocyclic organic groups. In the crystallooptical aspect, the *BZL* is an optically active uniaxial crystal with refractive indices $n_o$ = 1.663 and $n_e$ = 1.681 and a rotatory power $\rho =$ 24.3 deg/mm ($\lambda =$ 589 nm) along its optical axis^46^. The optical absorption spectrum of *BZL* in the bulk crystal phase is characterized by two absorption processes with energetic thresholds $E_{g1}$ = 2.84 eV and $E_{g2}$ = 3.55 eV that correspond to two split components of triplet $(n, \pi ^*)$-state^47^. The energetic threshold $E_{g1}$ is related to the absorption band at 3.25 eV (380 nm) which appears also in dilute solution of *BZL* in benzene thus it is associated with an intrinsic property of the *BZL* molecule and not with the exciton absorption. The emission band in *BZL*, located at 2.30 eV, is associated with the triplet phosphorescence emission from the $(n, \pi ^*)$ excited state. The large separation between the absorption and phosphorescence bands is associated with a significant conformational change between the ground state and triplet excited state configuration^47^. Recent nonlinear optical studies revealed that *BZL* crystals are characterized by a relatively high laser damage threshold (2.97 GW/cm$^2$ at 1064 nm) as well as by an enhanced saturation absorption and negative nonlinear refractive index^48,49^ which characterize it also as an efficient material for third order nonlinear optical applications. Taken together, *BZL* crystal can have favorable applications in laser frequency conversion such as e.g., second (SHG) and third (THG) harmonic generation. A sufficient optical transparency window in the visible region ensures a good optical transmission of SHG light induced, in particular, by Nd:YAG laser radiation^50^. Low values of the dielectric constant^51^, on the other hand, ensure in practice low power consumption and fast response time in relevant optoelectronic and photonic applications.

In terms of nanocomposite technology, *BZL* has significant technological advantage compared to its counterpart, trigonal quartz. Due to its low melting point (95 $^{\circ }$C^47^), benzil nanocrystals can be easily deposited into mesoporous silica from melt being capillary imbibed by nanochannels. Its solidification at cooling leads to the formation of preferred oriented crystals in pore space, i.e. nanocrystal texture. X-ray diffraction technique is used here to characterize the textural morphology of *PS*:*BZL* nanocomposites which appears to be dependent on the channel diameters of mesoporous silica. The SHG experiments aim to explore the second order nonlinear optical properties of *PS*:*BZL* composites. The spatial confinement reveals a significant influence on the nonlinear optical conversion efficiency in these nanocomposite materials.

## Experimental

The *p*SiO$_2$ substrates were prepared in two successive steps. In the first step, the channels were electrochemically etched into highly *p*-doped Si(100) wafers (resistivity 0.01$\div$0.02 ohm cm) using an electrolyte mixture of HF:C$_2$H$_5$OH (2:3) and dc current density 12 mA/cm$^2$. The resulting nanochannels are aligned perpendicular to the wafer surface, i.e., along the [100] crystallographic direction. In the second step, obtained in this way, the free-standing pSi membranes were subjected to a further thermal oxidation under standard atmosphere (12 h, *T* = 800 $^{\circ }$C). Using this technique, the obtained free standing optically transparent flowthrough mesoporous *PS* membranes have a typical view as presented in Fig. 1a. The porosity, *P*, the channel diameter, *D*, and the membrane thickness (the length of channels), *h*, of the electrochemically etched mesoporous silicon, *p*Si, likewise its oxidized counterpart, *PS*, rise with the etching time. Accordingly, by varying the etching time (2 $\div$ 8 h), we obtained the mesoporous *PS* membranes with average channel diameter *D* equal to 6.0 ± 0.4 nm (*P* = 12%, *h* = 100 $\upmu$m), 7.8 ± 0.6 nm (*P* = 21%, *h* = 160$\upmu$m), 9.4 ± 0.8 nm (*P* = 30%, *h* = 240 $\upmu$m) and 13.0 ± 1.0 nm (*P* = 49%, *h* = 310 $\upmu$m), as verified from volumetric N$_2$-sorption isotherms recorded at *T* = 77 K, hereafter referred to as *PS* (6.0 nm), *PS* (7.8 nm), *PS* (9.4 nm) and *PS* (13.0 nm) templates, respectively. Detailed description of the methodology used for characterization of *PS* matrices, including EDS analysis, desorption isotherms and pore size distribution (PSD) characteristics is provided in the Supplementary Information^52^. Typical SEM image of the obtained mesoporous *PS* substrates is shown in Fig. 1c.

Mesoporous *PS* templates were cleaned in a boiling acetone and ethanol ($\sim$ 1 h in each solvent) and then annealed at about 150 $^{\circ }$C for 1 h. The deposition procedure of BZL nanocrystals into silica nanochannels is sketched in sections (e) and (f) of Fig. 1. Dried *PS* templates were first completely filled with BZL melt via capillary imbibition at about 105 $^{\circ }$C. A further cooling (2 $^{\circ }$C/min) through the crystallization point ($T_{cr} < 95$ $^{\circ }$C) down to the room temperature leads to the formation of BZL nanocrystals inside the silica channels. One must mention that a considerable contraction of the confined BZL melt during its crystallization causes the formation of solidified bridges (crystalline nanoclusters) inside the silica nanochannels, separated by voids as schematically illustrated in Fig. 1f. That is why *PS*:BZL nanocomposite membranes considerably scatter both the reflecting and transmitting light. Relevant opaque scattering, absent in fully filled mesoporous membranes, becomes significant in even slightly underfilled substrates. A similar behavior has already been demonstrated in several very earlier studies, see e.g.,^53–55^. The textural morphology of synthesized silica–benzil nanocomposites is characterized with X-ray diffraction technique (DRON-4 diffractometer, CuK$_{\alpha }$ radiation) applying $\theta$-$2\theta$ angle scanning analysis ($\theta$-step of 0.01$^{\circ }$, exposure of 1 s).

Figure 1h sketches the nonlinear optical setup used for the measurements of the SHG response in the synthesized silica–benzil nanocomposites. It is build according to Maker fringe scheme and is similar to that used in the recent work^22^. Measurements at different incident (azimuthal) angles, $\alpha$, and polarization directions of the incident picosecond Nd:YAG laser light ($\lambda =$ 1064 nm, $E_p=$ 100 $\upmu$J, $\tau =$ 30 ps, repetition rate 10 Hz) aim at characterizing the eventual directional and light polarization anisotropy of SHG conversion efficiency in composite samples related particularly to the specificity of their textural morphology. For this reason, the samples were placed on the rotating optical stage controlled by a stepper motor. Azimuthal angles were scanned in one-degree steps with an integration time of 2 s, i.e., by averaging over 20 light pulses. The rotating half-wave plate serves to set a light polarization direction. Figure 1g describes the direction labels (*s*, *sp*, *p* or *ps*) corresponding to the four fixed light polarization directions explored in azimuthal SHG scans. Long focus lens L$_1$ is used here to set an appropriate (nondestructive) power density of the incident light on the sample. High-aperture short focus lens L$_2$ ensures an efficient collection of the fundamental and conversed light scattered by the sample in the solid angle of 1.5 sr. The SHG signal, selected by the interference filter F ($\lambda _F=$ 532 nm), is detected by a photomultiplier (PM) with a boxcar averager (integration time 0.1 s). The measured SHG signal of composite samples is compared to the SHG response of the reference sample, 1 mm-thick crystal quartz ($\alpha$-SiO$_2$) plate, measured in *ooo*-geometry ($\chi _{111}$-component of the second-order optical susceptibility tensor).

## Results and discussion

### Textural morphology

**Figure 2:**
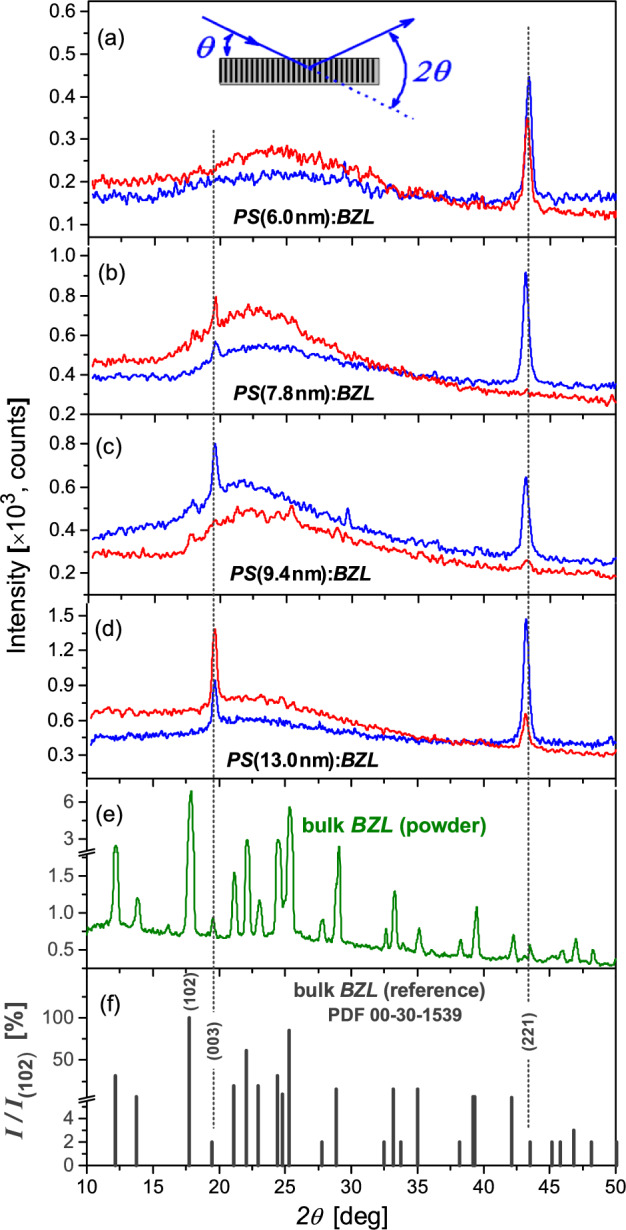
X-ray diffraction patterns ($\theta /2\theta$-scan) recorded from the opposite sides of nanocomposite *PS*:*BLZ* membranes: *PS* (6.0 nm):*BZL* (**a**), *PS* (7.8 nm):*BZL* (**b**), *PS* (9.4 nm):*BZL* (**c**) and *PS* (13.0 nm):*BZL* (**d**). (**e**) powder X-ray diffraction pattern of bulk *BZL*. (**f**) Reference powder X-ray diffraction pattern of *BZL*^56^. The inset in section (**a**) describes the X-ray diffraction geometry used in the characterization of textural morphology in nanocomposite *PS*:*BLZ* membranes.

The structure of nanocomposite materials, and especially their texture morphology, seems to be an important factor influencing the anisotropy of physical properties, including the nonlinear optical ones. We explore it using the X-ray diffraction (XRD) technique based on the diffraction geometry described in the inset of Fig. 2. In such a case, the crystalline atomic planes perpendicular to the long pore axis are probed. Basically, this makes sense taking into account that the elongated geometry of the channel enforces the nanocrystals to grow usually along certain crystallographic directions. Sections (a)–(d) of Fig. 2 present XRD patterns ($\theta /2\theta$-scans over the range 10$^{\circ }$–50$^{\circ }$) recorded from the opposite sides of nanocomposite membranes *PS* (6.0 nm):*BZL*, *PS* (7.8 nm):*BZL*, *PS* (9.4 nm):*BZL* and *PS* (13.0 nm):*BZL*, respectively. The recorded powder XRD pattern of bulk *BZL* (Fig. 2e) likewise the indexed reference powder diffractogram of *BZL* (Fig. 2f), taken from JCPDS database (PDF 00-30-1539^56^), are presented here for comparison. The positions of XRD peaks of the bulk *BZL* reveal good agreement with the JCPDS file.

The relative Bragg intensities in the recorded XRD patterns of *PS*:*BZL* nanocomposites (Fig. 2a–d) basically indicate *BZL* nanoclusters of two crystalline orientations with respect to the long channel axis, (221) and (003), wherein their ratio evidently depends on the pore diameter of the host *PS* substrate. Particularly, at small pore diameters, the nanocrystals of (221) orientations fully dominate, see e.g., *PS* (6.0 nm):*BZL* (Fig. 2a). Nanocrystals of (003) orientation were revealed only in *PS* substrates with larger pore diameters, wherein their fraction evidently rises with the channel size reaching almost parity ($\sim$45%) with nanocrystals of (221) orientation for pore diameters roughly larger than 13 nm, see Fig. 2d. One must mention that the XRD patterns recorded from the opposite sides of nanocomposite *PS*:*BLZ* membranes evidently differ, which indicates that the *BZL* texture is inhomogeneous along the nanochannels. This feature was also noticed in previous studies^24^ and among others can be attributed to a slightly conical shape of the nanochannels, which is known to occur during the electrochemical etching process of mesoporous silicon^22^.

Considering that the lateral size of *BZL* nanocrystals is restricted by the diameter of channels, the front of spontaneous crystallization may propagate along nanochannels over considerable distances. Extracting the full width at half maximum (FWHM) of XRD peaks (003) and (221) (Fig. 2) and applying the Scherrer equation^57^, one obtains practically the same average size, about 22 nm, for confined *BZL* nanoclusters along the long channel axis, i.e., it appears to be independent of the relevant crystallographic orientations. Accordingly, one can conclude that a spontaneous crystallization process in nanochannels is probably preceded by the formation of liquid phase capillary bridges separated by voids. Hence, spontaneous crystallization takes place individually in spatially separated capillary bridges resulting in the formation of nanoclusters of different crystallographic orientations, i.e., a nanocrystalline texture.

### SHG response

**Figure 3:**
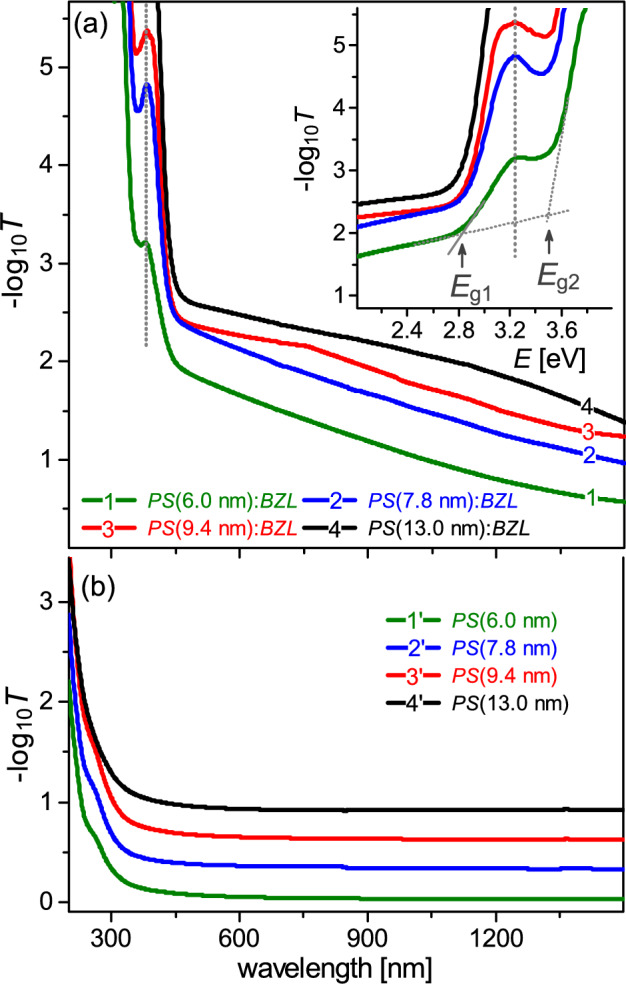
Comparative optical absorbance spectra, $-\log _{10}T$ vs $\lambda$, for series of *PS*:*BZL* nanocomposites [section (**a**)] and mesoporous *PS* host substrates [section (**b**)], see labeled. The inset [section (**a**)] details the absorption band of *PS*:*BZL* nanocomposites being assigned to dicarbonyl groups absorption of guest *BZL* component. The absorbance spectra of *PS* substrates, curves 2$^{\prime }$, 3$^{\prime }$ and 4$^{\prime }$ [section (**b**)], are sequentially shifted up by a step of 0.3 to avoid an overlapping of the curves.

Optical properties of PS:BZL composites are considerably dominated by its organic guest component. Figure 3 shows the UV-Vis optical absorbance spectra, $-\log _{10}T$ vs $\lambda$, for series of *PS*:*BZL* nanocomposites [section (a)] and mesoporous *PS* host substrates [section (b)] for comparison. Whereas the short-wave edge of optical transparency of empty *PS* membranes extends in UV rąnge down to 250 nm, for *PS*:*BZL* composites it is shifted to the visible region, $\sim$440 nm. The optical absorption process in *PS*:*BZL* composite, similarly to bulk *BZL*^47^, is characterized in this spectral range by two energetic thresholds at $E_{g1}$ = 2.82 eV and $E_{g2}$ = 3.51 eV, see marked in inset of Fig. 3a. The derived thresholds values appear to be very close to those of bulk crystal *BZL* reported by Stanculescu *et al*^47^, $E_{g1}$ = 2.84 eV and $E_{g2}$ = 3.55 eV, respectively. The inset in Fig. 3a details the first absorption band of *PS*:*BZL* nanocomposites being assigned to dicarbonyl absorption^58^ of guest *BZL* molecules. Its spectral location (382 nm) within experimental accuracy does not depend on the channel pore diameter of composite matrix coinciding practically with the spectral position of relevant absorption band in the bulk *BZL* (380 nm)^47^.

**Figure 4:**
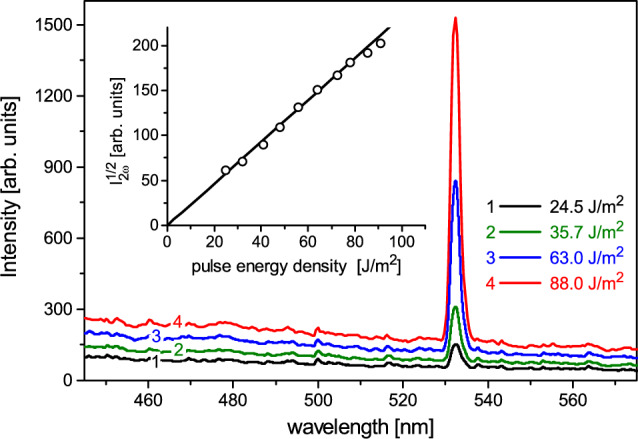
SHG emission by *PS* (13.0 nm):*BZL* nanocomposite. Curves 1–4 represent the spectra of the output SHG signal captured by CCD spectrometer for different magnitudes of laser pulse energy density (Nd:YAG laser, $\lambda =$ 1064 nm), see labeled. The inset presents the square root of SHG intensity, $I_{2\omega }^{1/2}$, vs the laser pulse energy.

Considering nonlinear optical behavior of a nanocomposite hybrid material one should also characterize the relevant properties of mesoporous host matrix. The oxygen nonstoichiometry of oxide materials indeed may play a crucial role in enhancement of nonlinear response efficiency in transparency range via resonant excitation of the deep defect states as has been demonstrated in several earlier studies^59,60^. The mentioned effect can modify degenerate cubic nonlinear optical response, promoting the parametric scattering process via highly polarizable excited states and possible pump laser radiation localization. However, it is applicable to the effects arising from a third-order optical nonlinearity, e.g. nonlinear optical focusing and defocusing, four wave mixing and particularly third harmonic generation (THG). The SHG effect, or optical frequency doubling, like any other even-order nonlinear optical phenomena, is forbidden in a media with inversion symmetry. Our measurements show that the SHG signal from empty mesoporous *PS*-membranes does not exceed instrumental noise level, i.e. it is at least of 10$^5$ times smaller than the SHG response of the reference crystal quartz sample ($\chi _{111}$-component). It confirms thereby an earlier suggestion of the disordered (amorphous) origin of *PS* matrix, in other words its intrinsic centrosymmetry regardless of Si/O stoichiometry deviation. However, in *PS*:*BZL* composite embodiment, the macroscopic inversion symmetry appears to be broken due to noncentrosymmetric trigonal structure of guest crystalline *BZL*-nanoclusters embedded into an amorphous *PS* scaffold. Such nanocomposites exhibit nonlinear optical emission. Characteristic spectra of the response signal recorded for different magnitudes of laser pulse energy density are shown in Fig. 4 for *PS* (13.0 nm):*BZL* nanocomposite as an example. Two experimental observations, i.e. the narrow peak at 532 nm and the quadratic variation of the collected signal vs the laser pulse energy clearly indicates its SHG origin. It is worth noting that the presented spectra do not exhibit the triplet phosphorescence band, which in principle could be excited by nonlinear optical harmonics of higher order, in particular by the third harmonic. Presumably their intensity is too low to induce noticeable phosphorescence emission.

Understanding and controlling the SHG effect in the relevant nanocomposite materials can lead to the development of novel nonlinear optical materials for an efficient laser light conversion, integrated optics and telecommunication applications. Here, the following two aspects become important. The first aspect is the anisotropy of the SHG effect regarding the direction of incident light and its polarization. The second aspect concerns the spatial confinement effect on the light conversion efficiency. For these reasons, nanocomposite *PS*:*BZL* membranes were azimuthally scanned over the $\alpha$-range from -60 to 60 degrees employing different polarization directions of the incident pump light, see SHG setup in Fig. 1h.

**Figure 5:**
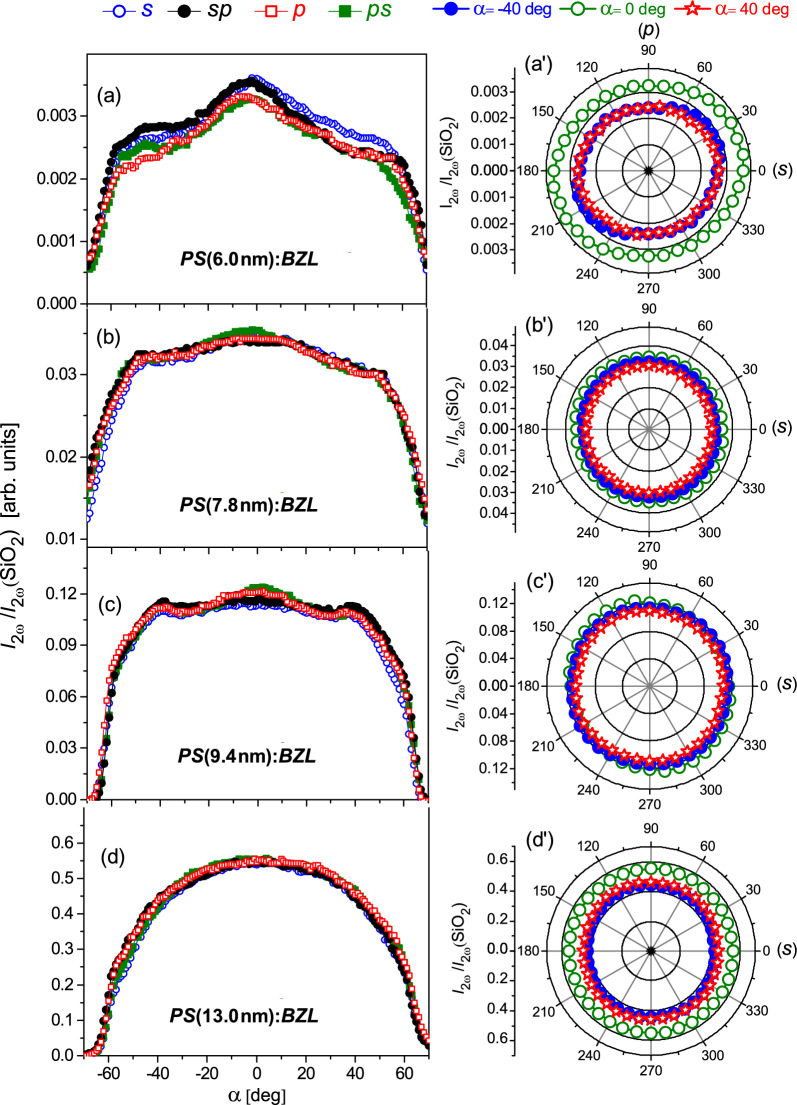
SHG response versus incident angle, $\alpha$, for four fixed light polarization directions *s*, *sp*, *p*, *ps* [left-hand panels (**a**–**d**)] and corresponding light polarization dependences of SHG response for three fixed incident angles $\alpha$ (− 40$^{\circ }$, 0$^{\circ }$ and 40$^{\circ }$) [right-hand panels (a$^{\prime }$–d$^{\prime }$)] measured in nanocomposite *PS*:*BLZ* membranes: *PS* (6.0 nm):*BZL* [(**a**) (a$^{\prime }$)], *PS* (7.8 nm):*BZL* [(**b**) (b$^{\prime }$)], *PS* (9.4 nm):*BZL* [(**c**) (c$^{\prime }$)] and *PS* (13.0 nm):*BZL* [(**d**) (d$^{\prime }$)]. The experimental error in the right-hand panels equals to the point size.

Left-hand panels of Fig. 5 display the SHG response versus the incident angle, $\alpha$, recorded for four fixed light polarization directions *s*, *sp*, *p*, *ps* in silica–benzil nanocomposite membranes: *PS* (6.0 nm):*BZL* (a), *PS* (7.8 nm):*BZL* (b), *PS* (9.4 nm):*BZL* (c) and *PS* (13.0 nm):*BZL* (d). Right-hand panels, (a$^{\prime }$–d$^{\prime }$) present the light polarization dependences of the SHG response measured at several fixed incident angles $\alpha$ (− 40$^{\circ }$, 0$^{\circ }$ and 40$^{\circ }$).

The SHG response from nanocomposite *PS*:*BZL* membranes generally shows a fairly broad angular dependence with no fringes and maximum conversion efficiency at normal incident geometry ($\alpha$=0$^{\circ }$). For smaller channel diameters, 6.0 - 9.4 nm (see Fig. 5a–c), these dependences are somewhat structured indicating a certain spatial anisotropy of SHG response caused presumably by a specific textural morphology of the embedded *BZL* nanoclusters. The analysis of spatial anisotropy appears to be a challenge due to the capillary voids causing a considerable light scattering as well as the light depolarization effects. In addition, textural morphology of *PS* (7.8 nm):*BZL*, *PS* (9.4 nm):*BZL* and *PS* (13.0 nm):*BZL* composites is dominated by crystalline nanoclusters of two crystallographical orientations, (003) and (221) (see Fig. 2b–d), wherein their lateral orientations are not fixed, and may be considered as random. For this reason, the SHG response curves recorded in these composite materials for different polarization directions of the incident pump light (*s*, *sp*, *p* or *ps*) overlap practically in most cases, see Fig. 5b–d. They appear to be consistent with the practically circular shape of relevant polarization dependences measured at several fixed incident angles, $\alpha = -40^{\circ }$, 0$^{\circ }$ and 40$^{\circ }$, see (Fig. 5b$^{\prime }$–d$^{\prime }$. In other words, the polarization anisotropy of SHG response appears to be completely reduced here due to the light depolarization on randomly oriented textural *BZL*-clusters. Composite *PS* (6.0 nm):*BZL*, by contrast, reveals a somewhat different behavior. A slightly elliptical shape of its angular polarization diagrams (Fig. 5a$^{\prime }$) actually indicates a weak but distinct light polarization anisotropy of the SHG conversion efficiency. However, one should be stressed that the composite morphology of this sample is characterized by a strong texture consisting of *BLZ* nanoclusters with only one crystallographic orientation, $\langle$221$\rangle$, parallel to the long channel axis (Fig. 2a). Amazingly, the SHG response reveals here a certain polarization anisotropy also in normal incident geometry ($\alpha =$ 0$^{\circ }$, Fig. 5a$^{\prime }$) indicating thereby that their lateral crystallographic orientations are not entirely random, i.e., presumably one deals with macroscopic quasi-single-crystal regions extending well beyond the size of single nanochannels. This may be caused by the specificity of the host *PS* medium, which is characterized by lateral interconnections between nanopores and is, therefore, properly described as 3D mesoporous medium rather than a set of independent tubular nanochannels. A propagation of the crystallization front through mesoporosity in the pore walls towards the neighboring pores probably causes the formation of large scale quasi-single-crystal regions evidenced in the SHG experiment. In fact, similar macroscopic single-crystal regions of dendrite shape were recently demonstrated in crystalline *PS*:*BNO* nanocomposites^24^.

**Figure 6:**
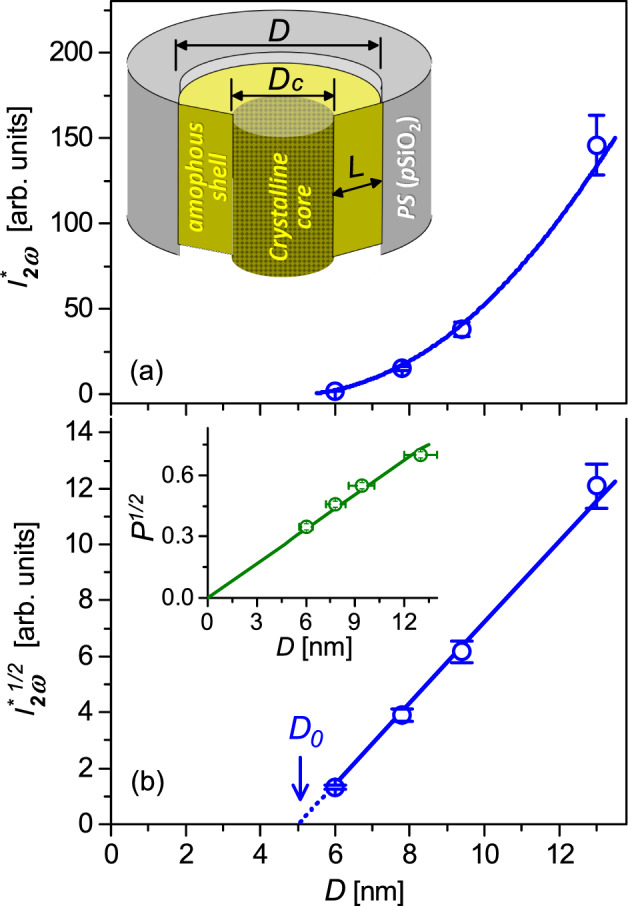
Normalized SHG response from nanocomposite *PS*:*BLZ* membranes, $$I_{2\omega }^*=I_{2\omega }h^{-1}k_t^{-1}/I_{2\omega } ($$SiO$$_2)$$ [section (**a**)] and its square root, $$I_{2\omega }^{*1/2}$$ [section (**b**)] versus the channel diameter *D*. Inset in section (**a**) sketches nanocrystalline cluster morphology consisting of amorphous disordered shell, covering the silica channel wall, and crystalline core. Inset in section (**b**) shows the dependence of square root of porosity $$P^{1/2}$$ on the channel diameter *D* for host *PS* membranes.

In nanocomposite *PS*:*BZL* media, the SHG light is generated exclusively by guest *BLZ* crystalline nanoclusters. The measured SHG intensity from such a multicluster system, similarly to the case of the colloid system^61,62^, can be related to the average crystalline cluster volume *V*, the number of nanocrystal clusters *N* appearing in the path of the incident laser beam, the effective second-order nonlinear polarizability $\langle \beta ^{(2)}\rangle$, transmission factor $k_t$ accounting for reduction of the conversed light due to the diffuse scattering and the fundamental light intensity $I_{\omega }$:

1 $$\begin{aligned} I_{2\omega } \propto k_tNV \langle \beta ^{(2)}\rangle ^2 I_{\omega }^2. \end{aligned}$$

The transmission factor $k_t$ depends on the composite morphology and membrane thickness *h*. By replacing in the experimental setup (Fig. 1h) the picosecond pulse Nd-YAG laser with the low power continuous wave laser ($\lambda =$ 532 nm) the transmission factor has been determined as the ratio between detected intensities with and without the sample in place. Relevant measurements give the $k_t$-value in the range of 0.01 - 0.02. One should admit therefore that capillary voids, as residues of a capillary crystallization process, cause a considerable multiple light scattering and presumably light depolarization. As a result, the effective SHG conversion efficiency of *PS*:*BZL* composites is reduced in measured samples by 50 to 100 times. The expected performance of uniformly filled composite membranes and the associated SHG intensity should indeed be normalized by the transmission factor. In particular, for normal incident pumping light ($\alpha =0^{\circ }$) one obtains estimated conversion efficiency factors of 0.18±0.02 (*PS* (6.0 nm):*BZL*), 2.4±0.2 (*PS* (7.8 nm):*BZL*), 9.2±0.6 (*PS* (9.4 nm):*BZL*) and 45.5±4.0 (*PS* (13.0 nm):*BZL*) compared to the reference crystal quartz sample (*ooo*-geometry, $\chi _{111}$-component). Therefore, it can be concluded that the performance of *PS*:*BZL* composites can be significantly improved by achieving uniform filling of *PS*-channels with organic guest. Moreover, for nanocomposites with a uniform orientation of embedded organic nanocrystals one may benefit also from nonlinear optical anisotropy by selecting optimal incident geometry and appropriate light polarization direction. The fabrication of uniformly filled nanocomposites remains, however, a technological challenge and will require further efforts. This appears to be beyond actual research and will be considered elsewhere.

The data presented above clearly indicate the extremely strong influence of spatial confinement on the second-order optical nonlinearity and the related SHG conversion efficiency. Our aim is to discuss this issue in detail, implying that understanding of such behavior and the corresponding mechanism seems to be important from both a fundamental and technological point of view. The SHG response normalized by the membrane thickness and transmission factor, $I_{2\omega }/(hk_t)$, serves here as an appropriate quantity enabling a proper exploration of the spatial confinement effect in composite *PS*:*BZL* samples. The porosity *P* of *p*SiO$_2$ matrices, on the other hand, nearly scales with the channel diameter squared ($P^{1/2} \sim D$, see Fig. 6b, inset), indicating that the number of channels per unit membrane area is practically the same in all host pSiO$_2$ membranes. This implies that the number of nanocrystal clusters *N*, appearing in the path of the incident laser beam, depends only on the membrane thickness *h*. The normalized SHG signal is expected to be proportional to the average cluster volume *V*, i.e., proportional to the channel diameter squared, $\sim D^2$. In this approach we assume that the effective second-order nonlinear polarizability $\langle \beta ^{(2)}\rangle$ is nearly the same in all the nanocomposite samples due to essentially disordered crystalline nanocluster texture which is actually confirmed by nearly isotropic angular dependence of the SHG response on the pump light polarization, see Fig. 5a$^{\prime }$–d$^{\prime }$.

Figure 6a shows the normalized SHG response of *PS*:*BZL* nanocomposites, $$I_{2\omega }^*=I_{2\omega }h^{-1}k_t^{-1}/I_{2\omega } ($$SiO$$_2)$$, averaged over four light polarization directions at normal incidence ($$\alpha =$$ 0$$^{\circ }$$), vs the channel diameter of their host *PS*-membranes. As one may see, the spatial confinement reveals a significant influence on their nonlinear optical conversion efficiency. The normalized SHG response rises nonlinearly with channel diameter by almost two order of magnitude. Though an accurate quantitative description of such a behavior seems to be quite challenging for the reasons mentioned above, the analysis of SHG response in the region of small diameters deserves special attention. $$I_{2\omega }^{*1/2}(D)$$-dependence indeed exhibits a linear behavior (Fig. 6b), although its extrapolation down to zero intensity $$I_{2\omega }^*=0$$ (see dot-line) intersects the horizontal axis at about $$D_0=$$ 5.0 nm, i.e., evidently differs from zero expected by Eq. 1. Such a behavior can be rationalized assuming that the embedded *BZL* clusters are actually not uniformly crystalline but are characterized by a more complex morphology consisting of SHG-inactive amorphous disordered shell that covers the channel wall and SHG-active crystalline core as sketched in the inset of Fig. 6a. In other words, from the symmetry point of view, only noncentrosymmetric crystalline core regions can contribute to the second order optical nonlinearity and consequently to the SHG effect. By this remark Eq. 1 should be modified as follows: $$I_{2\omega }^*\propto V_c \langle \beta ^{(2)}\rangle ^2 I_{\omega }^2 \propto D_c^2 \langle \beta ^{(2)}\rangle ^2 I_{\omega }^2$$, where $$V_c$$ and $$D_c$$ are the volume and the diameter of crystalline core, respectively. The amorphous shell has the thickness $$L=D_0/2\approx$$ 2.5 nm, i.e it consists of two to three molecular layers. Its formation, in the physicochemical aspect, is caused by the specificity of the host-guest interfacial interactions and the topography of the channel walls, particularly their roughness, and in the simplest approximation does not depend on the channel diameter or its geometry. Hence, the crystalline core diameter is defined as $$D_c=D-2L$$ ($$D\ge 2L$$), i.e. tends to zero at $$D \rightarrow 2L$$. Taken together, this explains why SHG response in *PS*:*BZL* nanocomposites vanishes already when the spatial cylindrical confinement approaches the diameters of a few layers of guest molecules deposited in nanochannels, as demonstrated by the experiment.

## Conclusion

In conclusion, we demonstrate here a series of organic–inorganic nanocomposites materials combining mesoporous *PS* and *BZL*-nanocrystals embedded to its tubular nanochannels. Our efforts aim to design novel, efficient nonlinear optical composite materials in which inactive amorphous host *PS*-matrix provides mechanical robustness, whereas nonlinear optical functionality results from specific properties of the deposited guest *BZL*-nanocrystals. The methodology of *PS*:*BZL*-composite fabrication is based on the capillary crystallization of *BZL*-melt while cooling of entirely filled composite membranes through the solidification point down to the room temperature. A considerable contraction of the * BZL* melt during its crystallization inside the silica nanochannels results in a formation of the texture consisting of nanocrystalline clusters separated by voids. Considering that the lateral size of *BZL* nanocrystals is restricted by the silica channel walls, diameter of which in our studies varies from 6 to 13 nm, the front of spontaneous crystallization may propagate along the nanochannels over much longer distances. X-ray measurements suggest the average length of the crystalline clusters of about 22 nm, which turns out to be practically independent of the channel diameter and their crystallographic orientations. The recorded XRD patterns in *PS*:*BZL* nanocomposites reveal the nanocrystal clusters of the two crystallographic orientations, (221) and (003), being parallel to the long channel axis, wherein their ratio significantly depends on the channel diameter. At small pore diameters, the nanocomposite texture is dominated by the (221) orientation of nanocrystalline clusters. However, the fraction of (003)-oriented clusters rises with the channel diameter reaching almost parity with a number of (221)-oriented clusters for channel diameters larger roughly than 13 nm.

The specificity of the textural morphology similarly to the spatial confinement significantly influences the nonlinear optical features of composite *PS*:*BZL* materials explored in the SHG experiment. At larger channel diameters ($D\ge 7.8$ nm), the coexistence of crystalline nanoclusters of (221) and (003) crystallographic orientations causes a quite complex textural morphology. The polarization anisotropy of the SHG response appears to be completely reduced apparently due to the multiple scattering and light depolarization on randomly distributed *BZL*-nanoclusters of different crystallographic orientations. Small but distinct polarization anisotropy of the SHG response is revealed, on the other hand, in a nanocrystalline texture grown in *PS*-channels of small diameters ($D =6.0\,$nm), which corroborates with a strong texture morphology of a relevant nanocomposite membrane. In addition, the SHG response exhibits here a weak polarization anisotropy for the normal incident geometry, which indicates that lateral crystallographic orientations of the embedded *BLZ*-nanocrystals in this nanocomposite are not entirely random. The embedded *BZL* nanocrystals presumably form large scale quasi-single crystal regions extending well beyond the size of the single nanochannels. Capillary voids, as residues of a capillary crystallization process, cause a considerable multiple light scattering and light depolarization which apparently reduces the SHG conversion efficiency. Their removal remains a technological challenge and will require further efforts to address this issue.

A spatial confinement reveals a significant influence on the nonlinear optical conversion efficiency of *PS*:*BZL*-composite membranes. The normalized SHG response rises nonlinearly by more than one order of magnitude as the channel diameter increases from 6.0 to 13.0 nm. It vanishes, on the other hand, when spatial cylindrical confinement approaches the diameters of few layers of guest molecules deposited in nanochannels. Such a behavior can be rationalized assuming that the embedded *BZL* clusters are actually not uniformly crystalline but are characterized by a more complex morphology consisting of a disordered amorphous shell covering the channel wall and well ordered crystalline core. Since the amorphous shell is nonlinearly optically inactive, only the noncentrosymmetric crystalline core regions can contribute to the second order optical nonlinearity and therefore to the SHG effect.

Taken together, understanding and controlling the textural morphology in inorganic–organic nanocrystalline composites as well as its relationships with nonlinear optical properties can lead to the development of novel efficient nonlinear optical materials for light energy conversion with prospective optoelectronic and photonic applications.

## Supplementary Information

Supplementary Information.

## Data Availability

All data generated or analyzed during this study are included in this published article [and its supplementary information file].
