# Supplementary material for: Second harmonic generation on crystalline organic nanoclusters under extreme nanoconfinement in functionalized silica–benzil composites
Source: Sci Rep. 2023 Jun 19;13:9943. doi: 10.1038/s41598-023-37147-4 (PMC10279710; doi:10.1038/s41598-023-37147-4)
Supplement: Supplementary file 1 — Supplementary Information. [file 41598_2023_37147_MOESM1_ESM.pdf]

Supplementary material to:

Second harmonic generation on crystalline organic nanoclusters under extreme nanoconfinement in functionalized silica-benzil composites

Houda El Karout, Yaroslav Shchur, Anatoliy Andrushchak, Bouchta Sahraoui, Robert Wielgosz, Olha Kityk, Jarosław Jędryka, Yurii Slyvka, and Andriy V. Kityk

1) Characterization of *PS* ($pSiO_2$) membranes:

To characterize the pore radii and pore size distribution (PSD) of mesoporous *PS* membranes the gas adsorption-desorption isotherm method has been used, see recorded desorption isotherms of nitrogen (N_2) at T=77 K in Fig. S1.

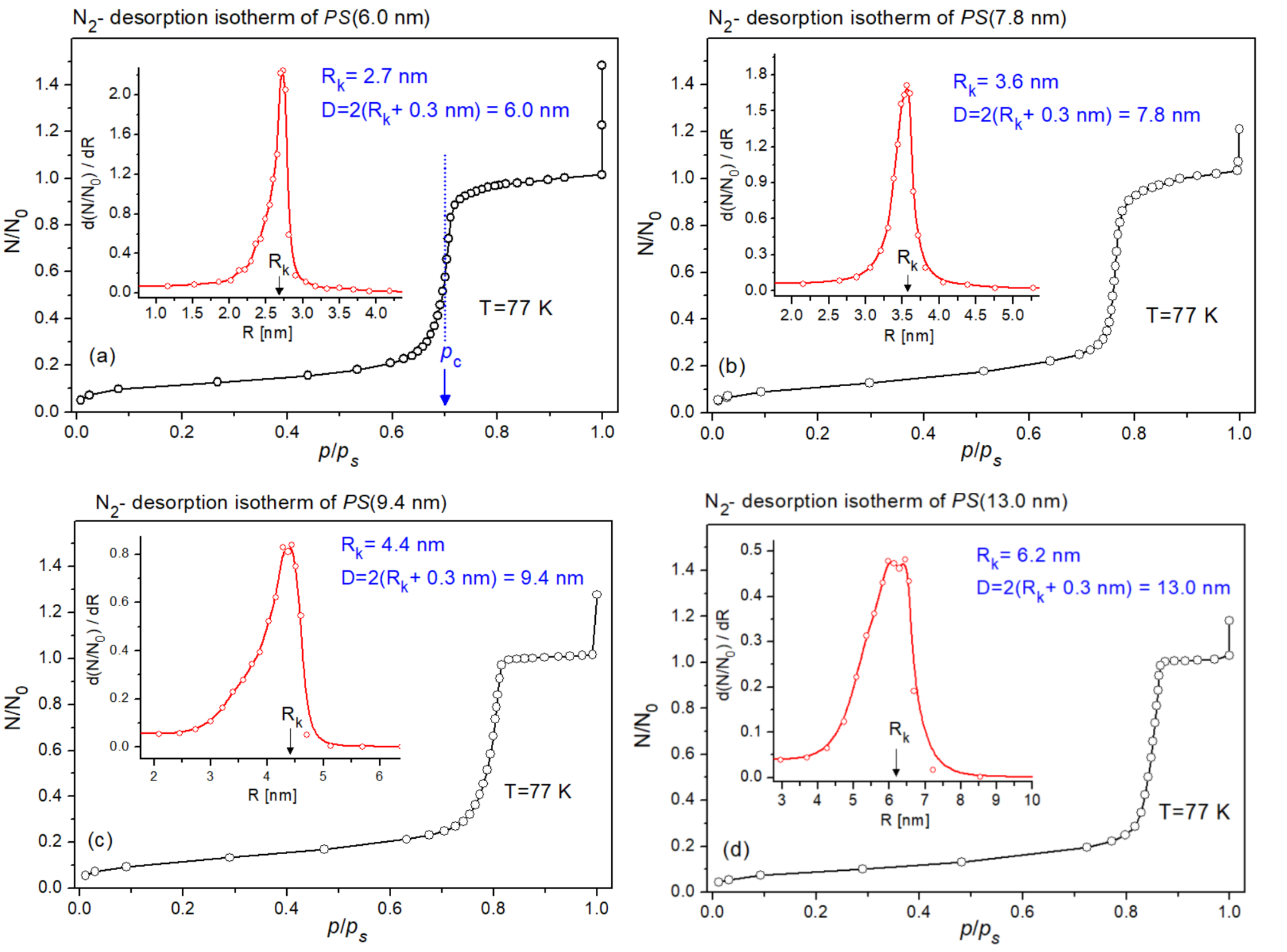

Figure S1. Desorption isotherms for mesoporous PS ($pSiO_2$) membranes. (a) PS(6.0 nm), (b) PS(7.8 nm), (c) PS(9.4 nm), (d) PS(13.0 nm). Insert shows pore size distribution (PSD).

The Kelvin radius R_k is related with capillary condensation pressure p_C by the Laplace-Kelvin equation:

$$R_k = \frac{2\gamma V_m}{R_0 T \cdot \ln(p_S\,/\,p_C)} \qquad (1)$$

where N/N_0 is the fraction filling, p_S and p_C are the saturated and pore condensation vapor pressures, respectively, V_m = 34.7 cm^3/mol is the molar volume of nitrogen, γ=8.85·10^{-3} N/m

is the surface tension, R_0 = 8.31 J/mol·K. Here R_k is so-called Kelvin radius approximately equals to the pore radius. More precisely, the pore radius $R = R_k + t_n$, where t_n is the thickness of the adsorbed wall layer, i.e. 1 - 2 molecular layers. For R ~ 3 - 5 nm t_n is often taken as 0.3 nm. Derivative d(N/N$_0$)/dR (see insets in Fig.1) characterizes the pore size distribution (PSD).

2) **EDS analysis of *PS*-membranes** (SEM microscope, Phenom ProX with EDS)

2.1. PS(6.0nm)

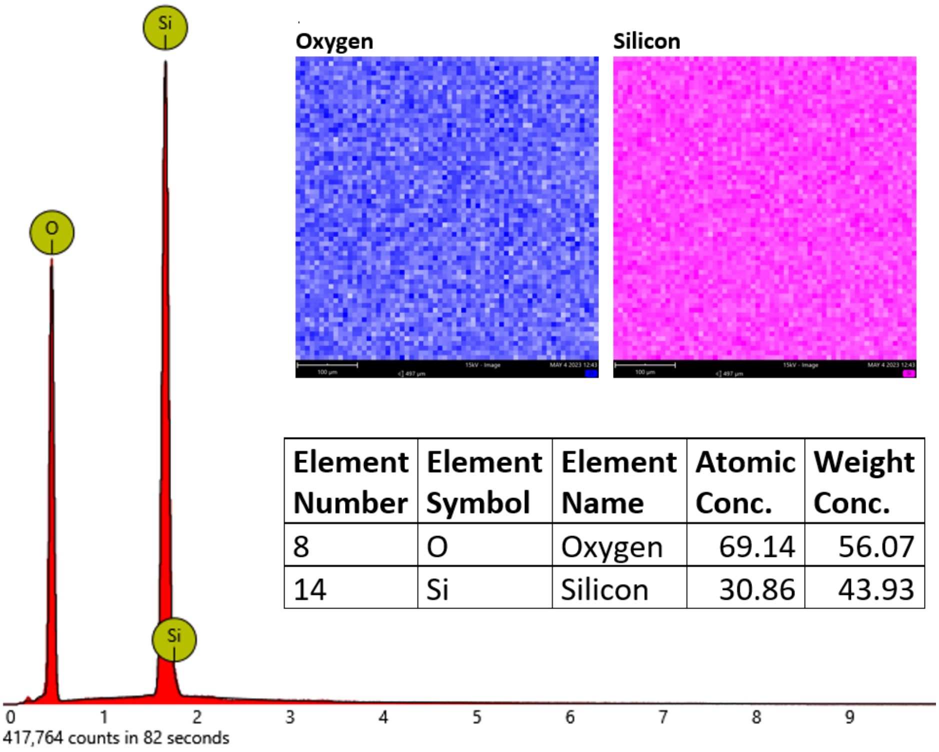

Element Number	Element Symbol	Element Name	Atomic Conc.	Weight Conc.
8	O	Oxygen	69.14	56.07
14	Si	Silicon	30.86	43.93

2.2. PS(7.8nm)

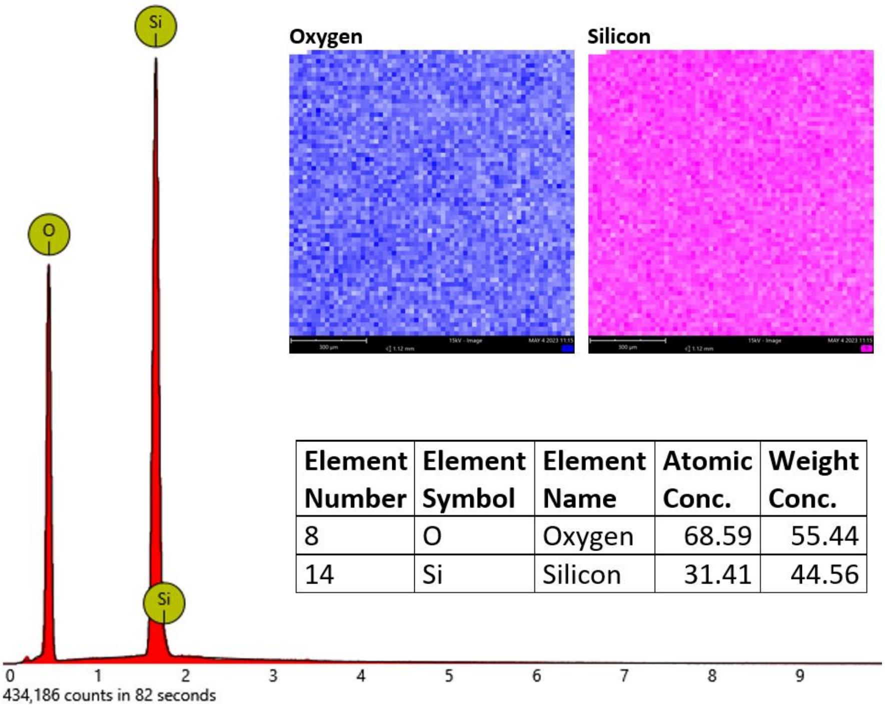

Element Number	Element Symbol	Element Name	Atomic Conc.	Weight Conc.
8	O	Oxygen	68.59	55.44
14	Si	Silicon	31.41	44.56

2.3. PS(9.4nm)

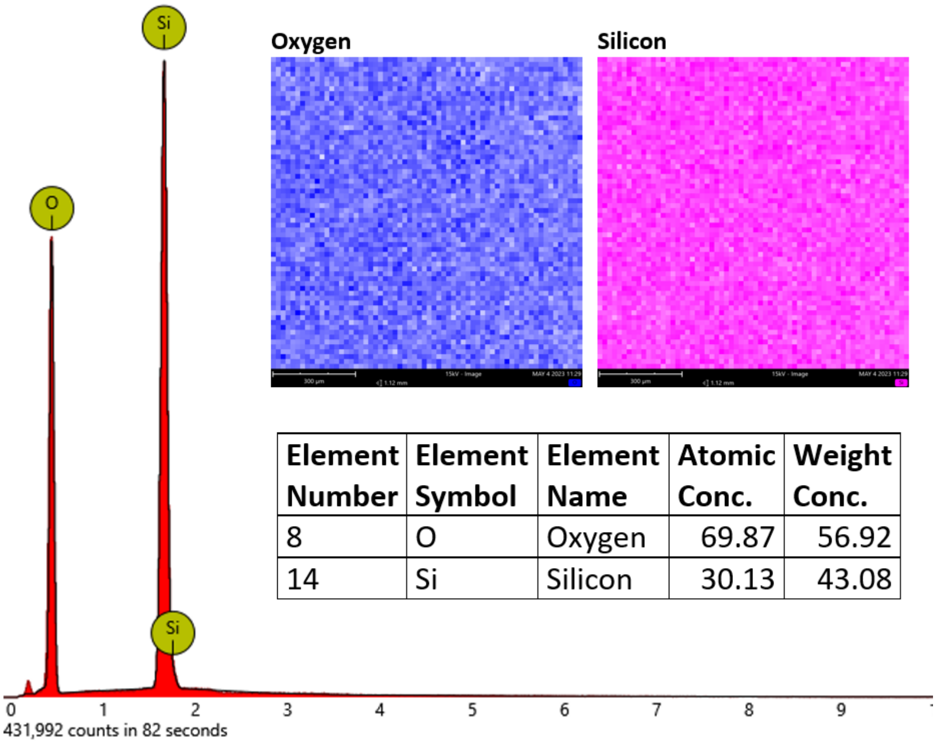

Element Number	Element Symbol	Element Name	Atomic Conc.	Weight Conc.
8	O	Oxygen	69.87	56.92
14	Si	Silicon	30.13	43.08

2.4. PS(13.0nm)

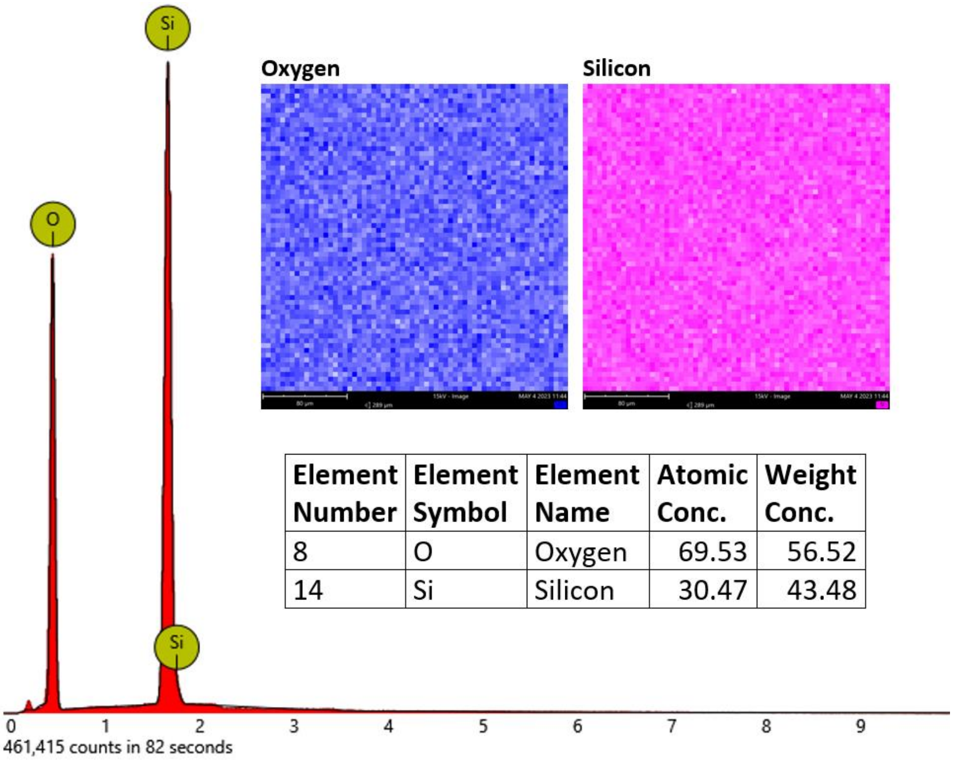

Element Number	Element Symbol	Element Name	Atomic Conc.	Weight Conc.
8	O	Oxygen	69.53	56.52
14	Si	Silicon	30.47	43.48
